# The effect of sowing time on the growth of chia (*Salvia hispanica* L.): What do nonlinear mixed models tell us about it?

**DOI:** 10.1371/journal.pone.0206582

**Published:** 2018-11-01

**Authors:** Diana Carolina Rodríguez-Abello, Jorge Augusto Navarro-Alberto, Luis Ramírez-Avilés, Roberto Zamora-Bustillos

**Affiliations:** 1 Departamento de Producción Animal en Agroecosistemas Tropicales, Facultad de Medicina Veterinaria y Zootecnia, Universidad Autónoma de Yucatán, Mérida, Yucatán, México; 2 Departamento de Ecología Tropical, Campus de Ciencias Biológicas y Agropecuarias, Universidad Autónoma de Yucatán, Mérida, Yucatán, México; 3 División de Estudios de Posgrado e Investigación, Instituto Tecnológico de Conkal, Conkal, Yucatán, México; Pennsylvania State University, UNITED STATES

## Abstract

Chia (*Salvia hispanica L*.) is an annual short-day plant whose growth has not been studied extensively in low-altitudes and at temperatures outside of its optimal range. The objective of this study was to describe the growth dynamics of a chia crop from an experimental plantation in south-east Mexico, on three different sowing dates. The chia grew at temperatures (18–37°C) and an altitude (9 m a.s.l.) outside of the recommended conditions (20–30°C, 500–1000 m a.s.l.). Three individual-plant responses were measured weekly, before seed harvest: height, number of leaves and number of inflorescences. Three theoretical nonlinear growth models were fitted to the data, a different model for each response. Mixed-effect model parameters were estimated by maximum likelihood, and the goodness of fit for each model was evaluated using two criteria: Modeling Efficiency and Root Mean Square Error. Chia seed yield was also measured in each treatment. Estimated parameters for plant height confirmed that medium sowing time (MST) and late sowing time (LST) plants had smaller heights than the early sowing time (EST) plants. Moreover, at the end of their life cycle, EST plants had a greater number of leaves and inflorescences, and higher seed yield. All of these differences were associated to the extended time of vegetative growth of EST plants favored by optimal photoperiod and temperature. Growth dynamics of chia during its ontogenic phases was explored, in more detail, with relative growth parameters derived from fitted models: a decrease in photoperiod influences the beginning of the reproductive phase, with the consequent reduction in speed of vegetative growth. In addition, nonlinear mixed-effects models can be useful in understanding the relation between growth parameters, plant maturity, and the suitable time for chia seed harvest. Our results suggest chia crops are adaptable to non-conventional environmental conditions.

## Introduction

The seeds of chia plants (*Salvia hispanica* L.) have been reported as being the vegetable source with the highest concentration of alpha-linolenic acid [1] and high in protein, fiber and antioxidants [2]. These properties have led to an increase in demand of seeds for direct consumption, oil, and processed foods, and subsequently an increase in production. Farmers have experimented with cultivating chia in various soil and climatic conditions and discovering the ideal sowing date for each cultivation area is a priority. The optimal conditions for chia growth are altitudes between 400 and 2,500 m a.s.l., average annual temperatures in the range of 20° to 30°C, and average annual rainfall between 500 and 1000 mm [3], and a floral induction photoperiod around 12:12 h [4]. Based on the measurements of morphometric characteristics throughout the vegetative cycle, several studies in low-altitude environments [5, 6, 7] describe the effect of sowing time on chia plant growth, emphasizing that photothermal conditions influence the development of the plants. However, these studies did not investigate how extreme temperatures affect chia growth. Therefore, our research question focuses on the analysis of chia growth for low-altitudes and temperatures outside the optimal range proposed [3]. It is expected that chia crops complete their growing cycle, under tropical conditions, outside their reported growth conditions, and their different developmental phases can be described using suitable growth models.

This study uses mathematical models to evaluate plant development from early stages until harvest, under changing phothermal conditions. In general, growth variables such as plant height, aerial biomass or main stem diameter are non-linearly related with time, and plants reach an asymptotic final size depending on the limitation of soil nutrients or changes such as the beginning of flowering [8]. Those nonlinear models may be particularly useful in characterizing plant height in chia plants [9, 10]. Other growth parameters can also be described by nonlinear models. Annual plants, chia included, have a unimodal behavior in plant parameters such as foliage cover [11] and number of leaves: after a rise period and reaching a maximum value, they decrease at the end of the growing season. Similarly, when selecting the model for the number of inflorescences over time, it is important to take into account that a decrease in photoperiod induces the onset of the reproductive phase in chia plants, and inflorescences finally emerge. Regularly, the rate of inflorescence emergence is a nonlinear increasing relationship that gradually slows down when reaching a maximum number.

The analyses of growth variables are strengthened by the benefits of the non-linear mixed modelling approach [12], where fixed effect factors (e.g. sowing time) and random effect factors (e.g. associated to individual plants) are incorporated into the models. Therefore, the main objective of this study was to describe the dynamics of growth variables of a chia (*S*. *hispanica*) seed crop using nonlinear mixed effects models for three responses: height, number of leaves per plant and number of inflorescences per plant, under non-conventional conditions of altitude, temperature and photoperiod. In particular, the modelling process evaluates how growth parameters are affected by varying sowing times.

## Materials and methods

### Field experiment and design

The experimental plantation consisted of three contiguous 8 x 10 m-plots, located in the northern part of the state of Yucatan, southeast Mexico, between 21° 01' 50" North latitude and 89° 29' 05" West longitude. The site has an average altitude of 9 m a.s.l., mean annual temperature of 26.5°C, average precipitation of 981 mm and a soil type classified as leptosol [13]. The study was conducted on private land with permission. Chia seed was sown during the rainy season of 2014, on the following dates: 1) 11 Aug. (EST); 2) 25 Aug. (MST); 3) 8 Sep. (LST). Plants sown on the same date occupied one randomly selected single plot. Within each plot, seeds were sown with 70 cm between rows; neither fertilizer nor agrochemicals were applied during the growth period. Supplementary irrigation was used to support the crop after three days without rainfall. Individual plants were considered as the experimental units of a completely randomized design with one fixed effect factor, the sowing date (identified in this study as “sowing time”). Fifteen plants were randomly selected from each experimental plot, and each plant was considered as a random effect in the model. During the growth period, from plant emergence to flowering, and before seed harvest, three responses were observed weekly, in 15 plants, randomly selected in each sowing time: plant height (cm), number of leaves and number of inflorescences per plant, as described in a previous work [14]. Data from plants that died during the experiment were removed from the analysis. The field study did not involve endangered or protected species.

### Growth models

The non-linear models used to describe the three referred responses of chia plants, are given in Table 1. For the final models chosen, their goodness of fit was investigated using basic graphs of observed data versus individual predictions [15], and two metrics commonly used in model performance evaluation [16]: Modeling Efficiency (MEF) and Root Mean Square Error (RMSE) [17].

**Table 1:** Functional expressions of the models used to describe growth of chia.

Variable	Model	No. of parameters	Equation	Reference
Height	Richards	4	*y* = *A*/(1+ *m* exp(–*K*(*t*– *λ*))^1/*m*^	[18]
	Logistic	3	*y* = *A*/(1+ *m* exp(–*K*(*t*– *λ*))	[19]
	Gompertz	3	*y* = *A* exp(–exp(–*K*(*t*– *λ*))	[20]
	von Bertalanffy	3	*y* = *A*(1 –exp(–*K*(*t*–*t*_0_))	[21]
Leaves	Double Richards	8	*L* = *A*/(1+ *m* exp(–*K*(*t*– *λ*))^1/*m*^ + *A*´/(1+ *m*´exp(–*K*´(*t*– *λ*´))^1/*m*´^	[22]
Inflorescences	Segmented	3	*F* = *φ* (1 –exp(–(*t*– *Λ*)/*μ*) **1**_(*Λ*,∞)_(*t*)	[23]

**Notation:** *y*, plant height (cm) at week *t*; *L*, number of leaves per plant at week *t*; *F*, number of inflorescences per plant at week *t*; *A*, asymptote for height / the first plateau for number of leaves; *A*´, difference between second and first curve plateaus for leaves; *K*, maturation rate for height; *K*´, rate of change between first and second plateaus for leaves; *λ*, inflection point for height / of the first curve for leaves; *λ*´, inflection point of the second curve for leaves; *m*, *m*´ = shape parameters producing changes in the inflection points and rates of change; *t*_0_ = adjustment for the initial height, giving the week at which plants would have had zero height; *Λ*, lag or resting time with no inflorescences; *φ* = peak parameter indicating the maximum number of inflorescences; *μ* = rate of change governing the steepness of the curve for the number of inflorescences, **1**_(*Λ*,∞)_(*t*) is the indicator function, taking the value 1 if *t* > *Λ*, and 0 otherwise.

### Model for plant height

The four-parameter Richards’ function [18, 24, 25] was considered as the maximal model for height *y*(*t*) of chia plants at week *t*. The chosen parametrization in the Richards’ curve (Table 1) follow an equation previously described [22] in the R-package FlexParamCurve [26]. However, the model comprised of both fixed and random effects for the asymptote *A*, the point of inflection *λ*, the rate of change *K* and the shape parameter *m* (S1 Model description), plus sowing time as an extra fixed-effect. The parsimony of this Richards’ model was evaluated by comparing its goodness of fit against the performance of three submodels of the Richards’ function, Logistic, Gompertz and von Bertalanffy models [9, 10, 27] (see also Table 1), by means of likelihood ratio tests [28]. The estimated fixed effects from the mixed model were used to calculate estimates of model parameters and the mean plant height for each sowing time; differences in the model parameters between pairs of sowing times were analyzed with *F* tests for contrasts.

The percentage of maturity, *PM*(t*) (S1 Model description), given as the percentage of the average asymptotic height reached at instant t*, was calculated for each sowing time at three separate instances: at the point of inflection, in the week when the first inflorescences emerged, and in the last week of plant height measurement, coinciding with seed harvest time. Average lifetime absolute growth rate (AGR), Average lifetime relative growth rate (RGR) and Average lifetime absolute maturing rate (AMR) were also calculated for each plant [29] (S1 Model description). AGR, RGR and AMR means were compared between sowing times using MANOVA and one-way ANOVAs, adjusting *P*-values for multiple testing via Bonferroni’s method. Significant differences of these growth rates for each pair of sowing times were evaluated with Tukey tests, with *α* = 0.05, under the condition of a significant one-way ANOVA for a particular growth rate. The instantaneous growth rate, *dŷ*/*dt* (cm week^–1^), was calculated for each plant, at three time periods, t* (S1 Model description): at the start of the flowering stage, at the estimated point of inflection, and at time of harvest, this latter considered as the time of seed maturity. Finally, the point of time delimiting the end of the lag phase of height, or Delta Value (in weeks), was estimated for each plant in every sowing time group (S1 Model description). Sowing times were compared with respect to the mean of the two instantaneous growth rates indicated above, and to the mean Delta Value, using generalized linear models and post-hoc Tukey’s tests, *α* = 0.05.

### Model for the number of leaves per plant

The number of leaves per plant *L*(*t*) was modelled as a function of time (*t* in weeks) using the Double Richards’ function [11, 22], denoted here as DRF. We remark that the model developed here is an approximation for the description of a discrete variable, using an expression for a continuous variable (S2 Model description). The DRF model takes into account that the number of chia leaves increases monotonically, to a turning point, indicating the start of the deciduous stage and leading to the loss of virtually all leaves. The Double-Richards’ function combines two Richards’ curves in one, comprising of up to eight parameters to be estimated. Four parameters control the first part of the curve, characterized by a raise in the response as *t* increases, up to a first (top) plateau; the remaining four parameters regulate the decreasing behaviour of the second part of the curve towards a second (bottom) plateau. All of the eight fixed-effect parameters in the DRF were considered for estimation, but only a suitable subset of the random effects were predicted, in order to assure convergence of the fitting process and interpretability of results (see details in the S2 Model description). Sowing date effects were added into the model as dummy variables, also for suitable growth parameters. Differences between pairs of sowing times for the referred subset of growth parameters were analyzed using *F* tests for contrasts, in a similar way to those comparisons carried out for plant height. All the calculations were executed in R [26], using algorithms implemented in the package FlexParamCurve [22].

### Model for the number of inflorescences per plant

The blooming dynamics of chia plants involves an early lag period without inflorescences followed by their blossoming and a subsequent stage of floral maturity process. This latter stage ended in the present study at seed harvesting. Therefore, the maximum number of inflorescences for each plant occurred close to the start of seed harvest. Accordingly, the model for the number of inflorescences *F*(*t*) at time *t* included a lag parameter (*Λ* in Table 1) specifying the duration of the resting time of chia plants with no inflorescences, and a function governing the monotonic increase in the number of inflorescences, up to a plateau (*φ*), before harvest. This behavior is captured in the non-linear segmented model [23] for *F*(*t*) shown in Table 1. We assumed that the segmented model is an approximation for the description of a discrete variable (number of inflorescences), using an expression for a continuous variable. Unlike the models for height, and number of leaves per plant, sowing time effect was not included in the model for *F*(*t*); this variable was modeled separately for each sowing time group, as the values of the resting time parameter, *Λ*, were assumed to be known *a priori* and not necessarily equal for all groups. The Mean Flowering Time (*MFT*), interpreted as the time needed for a plant to attain a number of inflorescences near the 63% of the asymptotic peak *φ*, was also calculated for each sowing time group. See S3 Model description, for details.

## Results

### Model for plant height

It was not possible to simplify the Richards’ model to consist of less than four parameters (Table 2). Therefore, the growth curves at each sowing time (S1 Model description) are characterized by the parameters of the Richards’ function presented in Table 3, highlighting a higher asymptote value (126.0 cm) for EST, significantly different from the corresponding asymptote values for MST (95.4 cm) and LST (94.0 cm). The rate of change (*K*) per week was lower (0.63) in the EST group with significant differences (*P* < 0.05) for LST (0.94), which attained the asymptotic size faster than EST; the MST group had an intermediate value (0.80).

**Table 2:** Likelihood ratio tests (LR) applied to compare Richards vs. Logistic, Gompertz and von Bertalanffy height growth models of chia plants.

Model	Df	Loglik	AIC	LR
Richards	23	−1550.3	3146.6	—
vs. Logistic	16	−1586.1	3204.3	71.7**
vs. Gompertz	16	−1712.5	3542.1	409.5**
vs. von Bertalanffy	16	−1730.8	3493.6	361.1**

** *P* < 0.0001 for a *χ*^2^ test with 7 degrees of freedom. Degrees of freedom (Df), the observed Log-likelihood (Loglik) and Akaike’s Information Criteria (AIC) for each model are shown. All models include the sowing time effect.

**Table 3:** Fixed effect estimates and variation of the random effects in the Richards height growth model.

Term	Fixed effect	Sowing time			Random effect SD
		Early	Medium	Late	
Asymptote, *A* (cm.)	Estimate	126.00^a^	95.44^b^	94.06^b^	20.18
	SE	5.50	5.63	5.64	
Rate of change, *K* (week^–1^)	Estimate	0.63^a^	0.80^ab^	0.94^b^	0.22
	SE	0.06	0.07	0.08	
Inflection point, *λ* (week)	Estimate	8.75^a^	7.36^b^	6.82^b^	0.76
	SE	0.22	0.22	0.23	
Shape, *m*	Estimate	1.28^a^	1.48^a^	1.91^a^	0.74
	SE	0.22	0.25	0.28	
Number of plants		14	13	13	Residual SD = 2.54

For each fixed effect, estimates followed by the same letter indicate non-significant differences for the corresponding contrast, using *F*-tests with *P* < 0.05

The EST plants with high asymptotic values and low rate of change (*K*) were less precocious in their growth than in other treatment groups, which can be corroborated by the low RGR values (0.276 vs 0.325 in average for MST and LST) and AMR (0.095 vs 0.118 in average for MST and LST). Despite those differences, the AGR was similar (*P* > 0.05) between sowing times (11.4 cm/week in average) (Table 4).

**Table 4:** Comparison of average lifetime chia growth rates by sowing time.

Average lifetime growth rate	Early		Medium		Late	
	Mean	SE (*n* = 14)	Mean	SE (*n* = 13)	Mean	SE (*n* = 13)
AGR*	11.95^a^	0.750	10.88^a^	0.640	11.30^a^	0.720
RGR*	0.276^a^	0.006	0.325^b^	0.006	0.326^b^	0.007
AMR*	0.095^a^	0.003	0.115^b^	0.003	0.120^b^	0.002

Wilks’ lambda = 0.250, *F* = 11.66, *P* < 0.0001.

*Average lifetime absolute (AGR, cm week^–1^), relative (RGR, cm week^–1^ cm^–1^ at inflection point) and maturity (AMR, cm week^–1^ cm^–1^ at asymptote) growth rates [29]. For each row, means followed by the same letter indicate non-significant differences, Tukey’s test (*P* < 0.05).

The inflection point (where the maximum growth rate is attained) in the EST group occurred at 8.75 weeks after sowing (WAS), being statistically different from MST (7.36 WAS) and LST (6.82 WAS) (Table 3). This point represented a proportion of the asymptotic size in the range from 52.5% to 57.1% (Table 5) and the instantaneous growth rate at this time was statistically similar for the 3 sowing groups (17.3 cm, in average). These similarities were also observed for the instantaneous growth rate at the start of the flowering stage, and at seed harvest (Table 6).

**Table 5:** Percentage of height maturity of chia plants in key times of growth.

Key time	Sowing time								
	Early			Medium			Late		
	Week	cm	%*	Week	Cm	%*	Week	Cm	%*
Inflection point	8.75	66.15	52.5	7.36	51.7	54.1	6.82	53.8	57.1
Inflorescence Emergence	10	88.06	69.9	8	62.2	65.2	7	56.9	60.5
Seed harvest^§^	16	124.72	99.0	14	95.0	99.5	12	93.9	99.9

*Percentage of maturity;

^§^ The last week of height measurement.

**Table 6:** Instantaneous growth rates at three particular times and delta values of height growth (Mean ±SE) of chia plants modelled by the Richards function, classified by sowing time.

Parameter	Sowing time		
	Early	Medium	Late
Instantaneous growth rate (cm week^–1^) At inflection point Start of the flowering stage At seed harvest (maturity)*	18.07^a^ ±1.16 15.42^a^ ±1.12 1.01^a^ ± 0.20	16.51^a^ ±0.94 15.39^a^ ±1.02 0.54^a^ ± 0.11	17.33^a^ ±1.07 16.22^a^ ±1.25 0.86^a^ ± 0.17
Delta Value (week)	5.10^b^ ±0.26	4.27^a^ ±0.16	3.74^a^ ±0.16

SE = Standard Error. For each row, means followed by the same letter indicate non-significant differences, Tukey’s test (P <0.05).

*Comparison of means and estimated standard errors computed via generalized linear modelling with gamma-distributed error and identity link. For the other parameters, the conventional one-way ANOVA with normal errors were applied.

**Fig 1:**
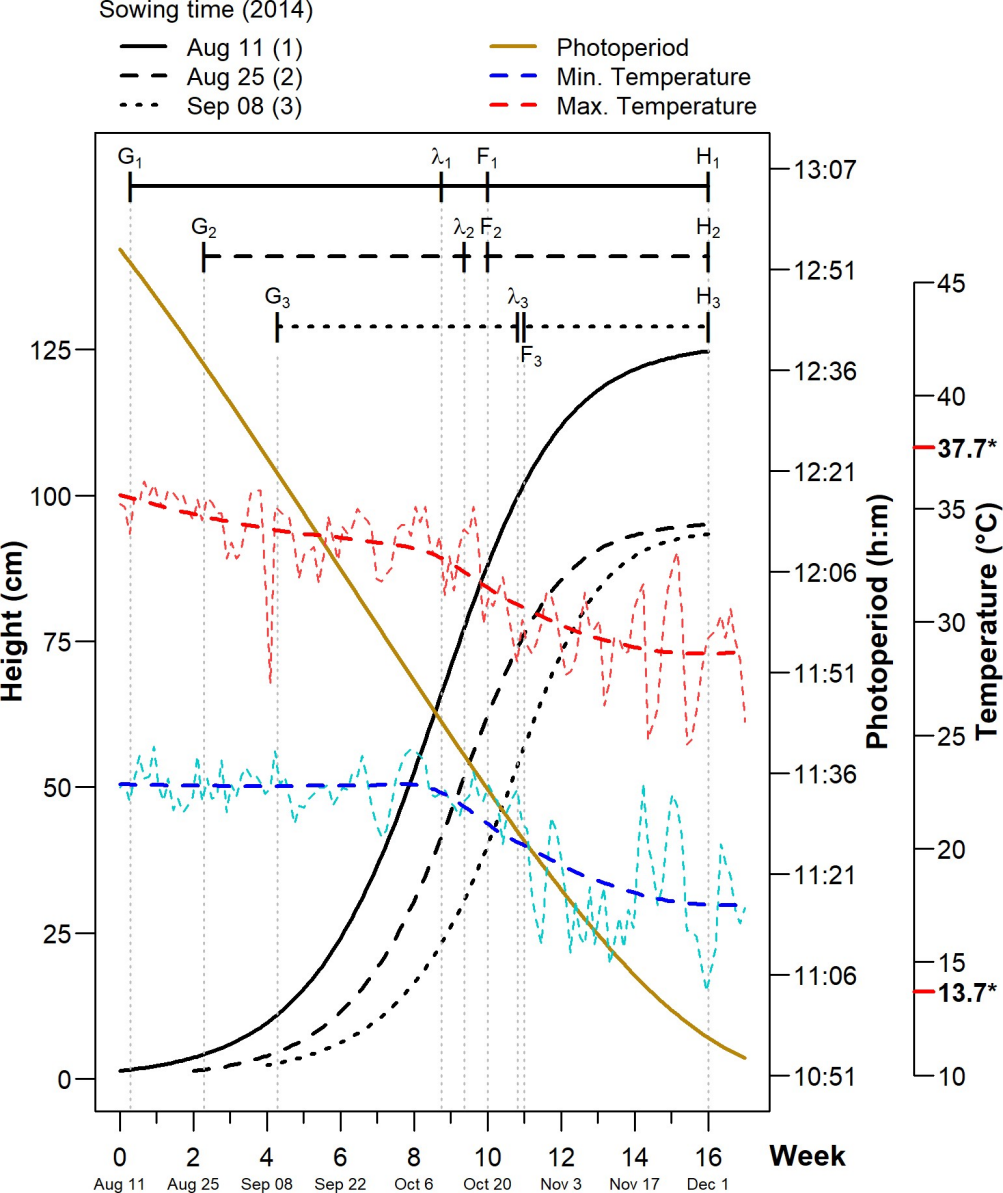
Fitted (sigmoid) Richards height models for three sowing times. The minimum and maximum weekly temperatures are displayed as two fluctuating time series (blue and red dashed lines), with their corresponding LOESS [30] trends superimposed. The decreasing line corresponds to the estimated photoperiod in the northern lowlands of Yucatan (meteorological station at the Autonomous University of Yucatan, Merida, Mexico). The phenological stages of chia plants are also shown: G = germination; *λ* = point of inflection; F = emergence of inflorescences; H = harvest. Numbers indicated with an asterisk next to the temperature axis indicate the minimum and maximum temperatures reached in 2014–2016, for the same study area and interval of weeks analyzed in the present investigation.

The fitted Richards’ curves seem to predict plant height accurately since the individual predictions follow the observations (S1 Fig). This is also supported by both graphical (Figure A in S1 Fig) and numerical procedures of model validation (Model Efficiency, MEF = 0.997, Root Mean Square Error, RMSE = 2.24 cm). On average, shapes of the fitted Richards’ curves were similar for the three sowing-time-groups (EST, MST and LST), but at different scales (Fig 1). This can be corroborated with the values of parameter *m*, which were similar between groups (Table 3). For all sowing times, plants reached full maturity at different times (16, 14 y 12 weeks for EST, MST and LST, respectively) (Table 4). The estimated delta point, indicating the end of the structuring (latency) phase, was 5.71 weeks in EST plants, while for plants in the MST y LST it was 4.27 and 3.74 weeks, respectively. In this phase, the instantaneous growth rate for EST was 5.10 cm week^–1^, being statistically different from MST (4.27 cm week^–1^) and LST (3.74 cm week^–1^) (Table 6).

### Model for the number of leaves per plant

Comparisons between sowing times in the number of leaves per plant, based on the fixed effect terms of the DRF, are presented in Table 7. The goodness of fit of the observed number of leaves versus the individual predictions (Figures B1, B2 in S1 Fig) were not as sound as the goodness of fit for height, but still the DRF is efficient (MEF = 0.976), and the average spread of the observed number of leaves around the predicted values is only RMSE = 5.52 leaves. The EST resulted, on average, in a higher number of leaves per plant (101.4, *P* < 0.0001) compared with the other treatments. The rates of change of the curve were similar among sowing groups (*P* > 0.05). Consequently, leaf emergence occurred at the same rate, with a maximum rate of leaf production observed in the weeks 8.92, 6.90 and 6.48, for EST, MST and LST, respectively.

**Table 7:** Comparison of the three sowing times according to selected fixed effect terms of the Double-Richards model for the number of leaves per plant.

Term	Sowing time			*F*_2,495_ (*P*)^**^
	Early^§^	Medium^§^	Late^§^	
*A* (number)	101.47^a^ (9.27)	39.87^b^ (9.90)	56.51^b^ (9.70)	11.12 (< 0.0001)
*K* (per week)	1.02^a^ (0.04)	1.04^a^ (0.10)	1.01^a^ (0.08)	0.038 (0.9626)
*λ* (week)	8.92^a^ (0.25)	6.90^b^ (0.33)	6.48^b^ (0.31)	22.65 (< 0.0001)
*m* = 1.87*	-	-	-	
*A´* (number)	–84.92^a^ (10.23)	–33.50^b^ (10.71)	–42.49^b^ (10.52)	6.92 (0.0011)
*K´* (per week) = 3.07*	-	-	-	
*λ*´ (week)	15.13^a^ (0.13)	12.40^b^ (0.18)	10.75^c^ (0.16)	233.50 (< 0.0001)
*m´* = 0.94*	-	-	-	

^§^Parameter estimates (Standard error in parentheses);

*Assumed equal for all three sowing dates, calculated as averages across all plants included in the three sowing times.

^**^ *F*-statistics and *P*-values of global tests of differences among sowing dates. On each row, different superscripts indicate significant differences between sowing dates (contrasts *F* tests, Bonferroni-corrected, *P* < 0.05).

The average value of *K*´ for the decreasing phase in the number of leaves was estimated at 3.07 week^–1^ (assumed equal for the three sowing dates in the modelling process). This shows a greater rate of leaf shedding in comparison to the leaf formation rate, the period when the maximum defoliation rate is attained (the inflection point, *λ*_*k*_´) as 15.1, 12.4 and 10.8 WAS for EST, MST and LST, respectively. All these aspects about the number of leaves are shown in the Figure B in S1 Fig.

### Model for the number of inflorescences per plant

The segmented model fit performed moderately for plants in the MST group and better for EST and LST groups, when used to predict the number of inflorescences once their corresponding resting time periods had ended (Figures C–E in S1 Fig) (see also the goodness of fit metrics (MEF and RMSE, shown in Table 8). Emergence of inflorescences occurred faster in the LST plants (7 WAS) in comparison to the EST plants (10 WAS). The mean flowering time (MFT) was similar (P>0.05) between EST and MST plants, but it was smaller for LST plants (P<0.05). Although LST group reached the flowering peak faster, it was much smaller than in the EST group (24.0 vs 9.0) (Table 8). This could explain the highest seed yield found, 330.3 vs 277.1 and 166.7 kg ha^–1^, respectively for EST, MST and LST groups (Table 9).

**Table 8:** Summary of estimated fixed and random effects in the fitted segmented models for the number of inflorescences per plant, with goodness of fit metrics.

Parameter	Description	Sowing time		
		Early	Medium	Late
Resting time, *Λ*	Constant (weeks)*	9	7	6
Peak, *φ* (No.)	Fixed effect estimate	24.0	8.3	9.0
	95% Conf. interval	(17.17, 30.8)	(5.36, 11.32)	(5.60, 12.48)
	Random effect SD	11.64	4.59	5.36
Steepness, *μ*	Fixed effect estimate	3.29	2.93	2.11
	95% Conf. interval	(2.81,3.76)	(2.27, 3.60)	(1.30, 2.92)
	Random Effect SD	0.13	0.31	1.08
MFT** (week)	Mean (SE)	12.29^a^ (0.01)	9.93^a^ (0.03)	8.11^b^ (0.07)
	Number of plants	12	10	10
MEF^§^	Modeling efficiency	0.936	0.874	0.925
RMSE^§^ (inflorescences)	Root mean square error	2.40	1.59	1.05
	95% Conf. interval	(2.09, 2.83)	(1.37, 1.91)	(0.89, 1.28)

Conf. interval: Confidence interval; SD: Standard deviation;

* Emergence of inflorescences occurred in the week after;

**Mean Flowering Time; SE: Standard Error;

^§^ Goodness of fit metrics.

**Table 9:** Life cycle and seed yield of chia sown in three different times.

Sowing time	Cycle (days)	Time elapsed from the start of blooming to seed maturing (weeks)*	Seed (kg/ha)**
Early	112	6	330.3
Medium	98	6	277.1
Late	84	5	166.7

* Calculated from Table 5, by subtracting the number of weeks elapsed, after sowing, until the first emergence of inflorescences to the number of weeks when seed harvest was carried out.

** Extrapolated from a sowing area of 80 m^2^.

## Discussion

### Model for plant height

The adjusted (sigmoid) Richards’ models for plant height used in the current study allows for the description of chia plant development. The model starts with a latency phase, in which the initial growth takes place, while germination and plant emergence occur; Delta values represent the end of this first phase. The second phase corresponds to the vegetative growth (known as the acceleration phase because the plant growth rate is high, and cellular hyperplasia and hypertrophy occur); in this phase, elongation and ramifications of the stem and leaf formation take place.

When the concavity of the growth curve changes at the inflection point, the deceleration phase starts, which coincides with the initial reproductive development (inflorescence formation and seed set). From this point onwards, growth speed is low until size at maturity or asymptote is achieved; this is a standing phase, matching with plant senescence. During this phase, leaf shedding occurs, after which seed harvest is carried out. The adjusted Richards’ models and the significant tests showed that the maximum (asymptotic) estimated plant height for the EST plants was higher and statistically different from the MST and LST. This could be due to the fact that EST plants had an extended life cycle (112 d vs 98 and 84 d for MST and LST plants, respectively), which was stimulated during the vegetative phase by the long photoperiod (from 12:10 to 12:55 h) and temperatures within a range from 23 to 36°C (Fig 1), considered optimal for chia growth [31]. This could increase photosynthetic rate and, consequently, there was an accumulation of photosynthates leading to a greater accumulation of plant tissue than other sowing times (MST and LST).

Early Sowing Time plants, with high asymptotic values and a low rate of change (*K*), have a longer vegetative phase than MST and LST, which reach an asymptotic size faster. This can be explained by the reduced vegetative growth (short juvenile phase) of MST and LST plants in comparison to EST plants which are required to achieve, in a short time, an appropriate maturity size, in order to start flowering as a response of a reduction on photoperiod. As a consequence, MST and LST grew faster than EST, the latter group requiring more time to undertake the same process; this is confirmed with the high RGR and AMR values. Delta values, an indicator of the latency phase, showed that this phase is longer in EST plants than in MST and LST plants, which confirms that the latter two were faster to reach mature size and reproductive phase than the former, in spite of being smaller in height. Also, MST and LST plants reached the inflection point sooner than the EST plants, as a consequence of their short vegetative phase, and they could not achieve the height of EST plants, although the rate of growth (17.3 cm/week) at this point was similar in the three sowing groups. From this point onwards, which coincides with the start of the reproductive phase, there was a gradual reduction on plant rate of growth until the asymptote was reached.

### Model for the number of leaves per plant

The asymptote for number of leaves in EST plants (101.47) was greater (*P* < 0.001) than those in the MST (39.87) and LST (56.51) groups, but there were no differences (*P* > 0.05) between the last two. As it was indicated previously, EST plants had a longer vegetative growth phase, when combined with appropriate temperature and light conditions, allowed stem elongation, and branch and leaf differentiation. Inflection points in the leaf growth curve (8.9, 6.9 and 6.5 WAS for EST, MST and LST, respectively) took place almost at the same time when the maximum plant height was recorded (8.8, 7.4 and 6.8 WAS, respectively for EST, MST and LST) showing allometric synchrony between plant height and leaf production.

The leaves’ senescence phase could last as long as the leaves’ maturation phase, since the senescence process involves changes that modify the structure and metabolism of leaves [32]. However, in the case of chia, it was observed that the senescence stage of the leaves occurred faster (*K*’ = 3.07 week^–1^ for the three sowing groups) than the formation and maturation (*K* = 1.2 week^–1^). This could be associated with the responses of the chia plants to the reduction of the photoperiod and the fact that it is an annual plant. As the photosynthetic process decreases, a reduction of nitrogen content of the chloroplasts is observed, which is related to the leaves yellowing and shedding [33].

Richard´s double function model allowed the description of the process of leaf loss (observed in the decreasing phase of the model) and the estimation of the number of leaves lost (obtained by the difference between asymptotes). Early Sowing Time and MST plants had 84% of leaves lost while LST had 75%. This process is an essential stage of plant development, since metabolic changes occurring in foliar tissues allow the recycling and remobilization of nutrients [34] that are used for the development of other organs, such as flowers and fruit [35]. Leaf shedding, besides being an indicator of maturity of the plant, facilitates the seed harvest [36].

### Model for the number of inflorescences per plant

Initial flower emergence was observed on the 20^th^ of October (2014), which corresponds to the 10^th^, 8^th^ and 6^th^ WAS of EST, MST and LST, respectively. However, only 40% of the LST plants had inflorescences on that date; therefore, the 7^th^, instead of the 6^th^ WAS was used for modelling, in order to accomplish the uniformity requirement for the three sowing times: to ensure that at least 75% of the plants in each group have one or more inflorescence.

The observation of the initial inflorescence emergence at the same date (20^th^ of October), in the three sowing times, indicates synchronicity of the chia crop in response to the environmental conditions. This synchronicity occurred in spite of inflorescence emergence in LST plants taking place earlier in the life cycle than in the EST and MST, implying that LST plants reached maturity at a younger age than EST and MST plants, according to a report made previously [8].

The maximum number (peak) of inflorescences was higher in EST than MST and LST plants, which explain the large differences on seed yield, 330.3, 277.1 and 166.7 kg/ha, respectively for EST, MST and LST. The difference in the number of inflorescences could be associated with the size of the plant (height); as suggested by previous work [8] that relates the high seed yield with a large accumulation of biomass.

Steepness and mean flowering time were very similar in all sowing times, an indication of the high level of synchronicity in inflorescence emergence. This is mainly due to the biological clock that makes plants flower in a synchronous manner as a response to climatic conditions, mainly photoperiod and temperature, provided that they have reached the appropriate mature size [37].

This study showed that chia is a plant sensitive to photoperiod, which is in agreement with the reports made by other authors [6, 7, 38]. Photoperiod at the experimental site (south-east Mexico) ranged from 12:55 h, in August, to 10:55, in December; this is a fluctuation of two hours of light during the life cycle, from sowing to seed harvest. The initial emergence of inflorescence took place when photoperiod was reduced, from 12:10 to 11:45 h, and air temperature ranged from 21 to 32°C. These climatic conditions favor chia floral induction, in accordance to literature reports [4, 39, 40].

It is important to point out that despite the fact that this is the first time that chia growth has been modelled, chia crops have been successfully harvested by other authors in the region (i.e. Yucatecan lowland) [3], despite the fact that apparently it is outside of its growing conditions. In the current study, we present data of only one year, which could limit its applicability in the future. However, if we consider that, in addition to day length and radiation, chia growth could be affected by air temperature (maximum and minimum), which were within the range intervals recorded during three years (Fig 1) It could be speculated that growth conditions were similar, and that chia plants would perform similarly within that range of climatic conditions considered as a typical year. Additionally, as indicated in the material and methods section, supplementary irrigation was supplied after three days with no rain, which prevented plant from suffering water stress. Therefore, although this study only reports results for one year, it could be expected that those would be applicable to any non-atypical year.

Several authors [6, 7, 38] evaluated the effect of sowing time on chia development, aiming at defining the optimal time of sowing for seed production. They found that early sowing allow greater plant vegetative growth and, consequently, higher seed yield, as was found in the current study. However, it is important to consider that sowing too early could result in larger plants with high risk of lodging; whereas very late sowing could prevent plants from reaching an appropriate size and physiological development in a such a way that seed production could be at risk.

## Conclusions

The cultivation of chia (vegetative and reproductive growth, and seed production) is feasible under the climatic conditions of Yucatan, southeast Mexico, with sowing date being the determinant factor of plant growth and seed yield. The adjusted Richards´ models for height and the number of leaves in conjunction with the segmented model for the number of inflorescences were useful to describe different stages in the growth of chia plants. The earlier the sowing is undertaken (with respect to the time of the year with short photoperiod) the taller plants are and the greater the number of leaves, inflorescences and seed yield will be. The estimated growth parameters indicate that a decrease in photoperiod influences the beginning of the reproductive phase of the plants, with the consequent reduction of speed of growth. Additionally, the estimated relative growth parameters derived from the parameters of the nonlinear mixed-effects models are valuable in determining the time of plant maturity and the suitable time for chia seed harvest.

## Supporting information

S1 Model description Richards’ model for height and derived parameters. (DOC)

S2 Model description Double Richards’ mixed-effects model for the number of leaves per plant. (DOC)

S3 Model description Segmented nonlinear mixed-effects model for the number of inflorescences per plant. (DOC)

S1 Fig Plots of fitted and predicted responses of chia plants using nonlinear mixed-effects models. (DOC)
