# Supplementary material for: The effect of sowing time on the growth of chia (Salvia hispanica L.): What do nonlinear mixed models tell us about it?
Source: PLoS One. 2018 Nov 1;13(11):e0206582. doi: 10.1371/journal.pone.0206582 (PMC6211711; doi:10.1371/journal.pone.0206582)
Supplement: S1 Model description — (DOC) [file pone.0206582.s001.doc]

**Model description: Richards’ model for height and derived parameters**

**Mixed-effects Richards height growth model**

The parameterization of the Richards function chosen in this study follows the mathematical expression given by Oswald *et al.* (2012) in the R-package FlexParamCurve (R Core Team, 2017), but it was extended to include random effects for each growth parameter and a fixed sowing period effect. The (four-parameter) Richards model was:

$y_{ik}(t_{k})=\frac{A_{ik}}{\left[1+m_{ik}\exp\left[-K_{ik}\left(t_{k}-\lambda_{ik}\right)\right]\right]^{\frac{1}{m_{ik}}}}+\varepsilon_{ik}, i=1,...,s_{k}, 0\leq t_{k}\leq T_{k}, k=1,2,3,$ (1)

where $y_{ik}(t_{k})$ = height (cm) of plant *i* at week *t_k_* sown at time *k*;*, s_k_* = number of plants (subjects) sown in the *k*-th time; *T_k_* is the maximum time (week) where plant height measurements were made for the sowing-period *k*. The Richards growth parameters, *A_ik_*, *K_ik_*, *λ_ik_* and *m_ik_*, determine the asymptote, the growth rate, the inflection point and the curve shape parameter, respectively, while $\varepsilon_{ik}\sim N(0,\sigma_{k}^{2})$is the random error term. All these parameters are summarized in the vector $\mathbf{\Phi}_{ik}$:

$\mathbf{\Phi}_{ik}=\left[\begin{matrix} A_{ik} \\ K_{ik} \\ \lambda_{ik} \\ m_{ik} \end{matrix}\right]=\left[\begin{matrix} \beta_{A_{1}} \\ \beta_{K_{1}} \\ \beta_{\lambda_{1}} \\ \beta_{m_{1}} \end{matrix}\right]+\left[\begin{matrix} \alpha_{2} & \alpha_{3} \\ \gamma_{2} & \gamma_{3} \\ \delta_{2} & \delta_{3} \\ \eta_{2} & \eta_{3} \end{matrix}\right]\left[\begin{matrix} x_{i2} \\ x_{i3} \end{matrix}\right]+\left[\begin{matrix} b_{A_{ik}} \\ b_{K_{ik}} \\ b_{\lambda_{ik}} \\ b_{m_{ik}} \end{matrix}\right] =\mathbf{\beta}_{1}+\mathbf{\Lambda}_{k}\mathbf{x}_{ik}+\mathbf{b}_{ik}$, $\mathbf{b}_{ik}\sim N(\mathbf{0},\Psi_{k})$ (2)

where $x_{ki}=\left\{\begin{matrix} 1 , & \text{Plant }i\text{ was sown at time }k \\ 0 , & \text{otherwise} \end{matrix} \right., k=2,3$. The *β*’s represent the four average Richards parameters for the first sowing time. The element $\alpha_{2}$ in the first row of the matrix $\mathbf{\Lambda}_{k}$ is the fixed-effect contrast of asymptotes for sowing times 1 (EST) and 2 (MST), ($\beta_{A_{2}}-\beta_{A_{1}}$), whereas $\alpha_{3}$ is the fixed-effect contrast of asymptotes for sowing times 1 (EST) and 3 (LST), $(\beta_{A_{3}}-\beta_{A_{1}})$. This parameterization for the product $\mathbf{\Lambda}_{k}\mathbf{x}_{ik}$ in (2) is the same as that used in the R-packages nlme (Pinheiro and Bates, 2000) and FlexParamCurve (Oswald *et al.* 2012) whenever a single fixed-effect covariate is included in a mixed effect model. The random-effects vector (**b***_ik_*), represent a deviation of the Richards function parameters (**Φ***_ik_*) from their population mean. These random effects are assumed independent for different plants and sowing times, and the random errors (*ε_ijk_*) are mutually independent for different *i*’s, as well as being independent from the random effects. As it was indicated in the Results section, none of the Richards’ submodels (Gompertz, Logistic and von Bertalanffy) improved the fit achieved by the Richards function itself. Therefore, the fitted height $\hat{y}_{k}(t)$for sowing time group *k* was computed from the fixed-effect Richards model; the sigmoid curves displayed in Figure 1 are the graphs for those fitted models.

**Percentage of maturity**

Equation 3 below gives the *percentage of maturity in the key instant t**:

. (3)

$PM_{k}(t_{k}*)$ is interpreted as the percentage of the average asymptotic height reached at instant $t_{k}*$ by plants sown in time *k*. The key instants considered in this study were: 1) at the point of inflection, 2) in the week when the first inflorescences emerged, and 3) in the last week of height measurement.

**Average Lifetime Growth Rates (Richards 1959; Perotto *et al*. 1992)**

The fixed and random-effects for *A, K* and *m* of the Richards model (1) were used to calculate the average lifetime absolute (*AGR*), relative (*RGR*), and maturity (*AMR*) growth rates (Richards, 1959). The three expressions shown in (4), are adjustments made to the original equations given by Perotto *et al.* (1992), considering the parameterization (1) of the Richards function and assuming that, for a given sowing time *k*, each average growth rate can be computed using the fitted growth parameters for each plant *i*:

; ; (4)

The computed *AGR*, *RGF* and *AMR* values were seen as realizations of a trivariate random variable classified by sowing time; the trivariate means of sowing times were compared through a one-way MANOVA; the assumptions of multivariate normality and homogeneity of the covariance between groups for the residuals were checked with Royston’s and Box’s M tests, respectively. If the MANOVA detected differences between sowing times, comparisons had to go further in order to detect univariate differences, this time with a one-way ANOVA. Post hoc Tukey tests were finally applied for those significant global *F* tests in the one-way ANOVAs. The significance level for all the tests was *α*=0.05, with the exception to the univariate ANOVAs, whose corresponding *p*-values were adjusted for multiple testing via Bonferroni’s method.

**Instantaneous rate of change of height for the Richards function**

It can be shown that the estimated instantaneous growth rate of the fitted height for plant *i* at time *t*, modelled by the Richards function (1), is:

(5)

For each plant in sowing time group *k*, this instantaneous growth rate (cm week^–1^) was calculated at two particular instants of time $t=t_{k}*$ : at the start of the flowering stage, $F_{k}$, and at the estimated point of inflection, $\hat{\lambda}_{k}$. Sowing times were compared with respect to these mean growth rates at particular instants of time, using one-way ANOVAs and post-hoc Tukey’s tests, with *α*=0.05.

**Delta Value or Lag phase of growth for plant height**

This parameter indicates the point of time delimiting the end of the lag phase of growth, for a particular plant modelled with a sigmoid curve $f(t)$ (e.g. Richards). An estimate of the Delta Value $\hat{\delta}_{ik}$ (in weeks) for the height of the *i*-th plant, belonging to the *k*-th sowing time and fitted with the Richards function (equation 1), is calculated as the *x­-*intercept of the tangent line to the Richards curve at the point of inflection ($\hat{\lambda}_{ik}$,):

, (6)

where is given by equation (5). Sowing times were compared with respect to their mean Delta Value using one-way ANOVA and post-hoc Tukey’s tests, *α*=0.05.

**References**

Oswald, S.A., Nisbet, I.C.T, Chiaradia, A., Arnold, J.M., 2012. FlexParamCurve: R package for flexible fitting of nonlinear parametric curves. Methods Ecol. Evol. 3: 1073-1077.

Perotto, D., Cue, R.I., Lee, A.J., 1992. Comparison of nonlinear functions for describing the growth curve of the three genotypes of dairy cattle. Can. J. Anim. Sci. 72: 773-782.

Pinheiro, J.C., Bates, D., 2000. Mixed-Effects Models in S and S-PLUS. Springer: New York

R Core Team. 2017. R: A language and environment for statistical computing. R Foundation for Statistical Computing, Vienna, Austria.

Richards, F.J., 1959. A Flexible Growth Function for Empirical Use. ‎J. Exp. Bot. 10:290-301.
