# Supplementary material for: The effect of sowing time on the growth of chia (Salvia hispanica L.): What do nonlinear mixed models tell us about it?
Source: PLoS One. 2018 Nov 1;13(11):e0206582. doi: 10.1371/journal.pone.0206582 (PMC6211711; doi:10.1371/journal.pone.0206582)
Supplement: S2 Model description — (DOC) [file pone.0206582.s002.doc]

**Model description:Double Richards mixed-effects model for the number of leaves per plant**

The Double Richards function (DRF) for $L_{ik}(t_{k})$, the number of leaves per plant *i* at time $t_{k}$ (week) for plants sown at time *k*, is (Oswald *et al*., 2012; Werker and Jaggard, 1997):

$\begin{matrix} L_{ik}(t_{k})=\frac{A_{ik}}{\left[1+m_{ik}\exp\left[-K_{ik}\left(t_{k}-\lambda_{ik}\right)\right]\right]^{\frac{1}{m_{ik}}}}+\frac{A'_{ik}}{\left[1+m'_{ik}\exp\left[-K'_{ik}\left(t_{k}-\lambda'_{ik}\right)\right]\right]^{\frac{1}{m'_{ik}}}}+\varepsilon_{ik}, \\ i=1,...,s_{k}, 0\leq t_{k}\leq T_{k}, k=1,2,3, \varepsilon_{ik}\sim N(0,\sigma_{k}^{2}). \end{matrix}$ (1)

It is assumed that $L_{ik}$ is an approximation of the number of leaves, a discrete variable, using a continuous random variable. The Richards function (1) combines two Richards curves in one, this is why it is called the “Double-Richards model”. The first curve increases as *t* increases, up to a plateau, and then the second curve decreases towards a second plateau. The parameters of the first curve are: $A_{ik}$(the first curve plateau), $K_{ik}$(the rate of change between the initial number of leaves and the first plateau), $\lambda_{ik}$ (the point of inflection of the first curve), $m_{ik}$ (the shape parameter of the first curve). Analogously, the remaining four parameters influence the behavior of the “decreasing stage”: $A'_{ik}$(the difference between plateaus of the first and the second curves), $K'_{ik}$(the rate of change between the first and second plateaus), $\lambda'_{ik}$ (the point of inflection of the second curve) and $m'_{ik}$ (the shape parameter of the second curve). All the fixed-effect parameters in the DRF were considered for estimation but only a suitable subset of the random effects were predicted, in order to assure convergence of the fitting process and interpretability of results. The chosen decomposition of fixed and random effects for the eight parameters were:

$\left[\begin{matrix} A_{ik} \\ K_{ik} \\ \lambda_{ik} \\ m_{ik} \\ A'_{ik} \\ K'_{ik} \\ \lambda'_{ik} \\ m'_{ik} \end{matrix}\right]=\left[\begin{matrix} \beta_{A_{1}} \\ \beta_{K_{1}} \\ \beta_{\lambda_{1}} \\ \overline{\beta}_{m} \\ \beta_{A'_{1}} \\ \overline{\beta}_{K'} \\ \beta_{\lambda'} \\ \overline{\beta}_{m'} \end{matrix}\right]+\left[\begin{matrix} \alpha_{2} & \alpha_{3} \\ \gamma_{2} & \gamma_{3} \\ \delta_{2} & \delta_{3} \\ 0 & 0 \\ \eta_{2} & \eta_{3} \\ 0 & 0 \\ \omega_{2} & \omega_{3} \\ 0 & 0 \end{matrix}\right]\left[\begin{matrix} x_{i2} \\ x_{i3} \end{matrix}\right]+\left[\begin{matrix} b_{A_{ik}} \\ 0 \\ b_{\lambda_{ik}} \\ 0 \\ b_{A'_{ik}} \\ 0 \\ b_{\lambda'_{ik}} \\ 0 \end{matrix}\right]$. (2)

The decomposition (2) indicates that we only estimated the fixed effects and predicted the random effects for $A$, $A'$, the two inflection points, $\lambda$ and $\lambda'$, while just the fixed effect for the first curve, *K*, was estimated. The bars over the$\beta$’s indicate that the estimated fixed effects for *m*, $K'$ y $m'$ were calculated as means throughout all plants included in all the three sowing times. This means that we did not make any distinction between sowing times for *m*, $K'$ y $m'$. All the calculations were executed in R (R Core Team, 2017), using algorithms implemented in the package FlexParamCurve (Oswald *et al.*, 2012). Differences between pairs of sowing times for the remaining five parameters were analyzed using *F* tests for contrasts, similarly to those comparisons carried out for height.

**References**

Oswald, S.A., Nisbet, I.C.T, Chiaradia, A., Arnold, J.M., 2012. FlexParamCurve: R package for flexible fitting of nonlinear parametric curves. Methods Ecol. Evol. 3: 1073-1077.

R Core Team. 2017. R: A language and environment for statistical computing. R Foundation for Statistical Computing, Vienna, Austria.

Werker, A.R., Jaggard, K.W., 1997. Modelling asymmetrical growth curves that rise and then fall: applications to foliage dynamics of sugar beet (*Beta vulgaris* L.). Ann. Bot. 79: 657-665.
