# Supplementary material for: The effect of sowing time on the growth of chia (Salvia hispanica L.): What do nonlinear mixed models tell us about it?
Source: PLoS One. 2018 Nov 1;13(11):e0206582. doi: 10.1371/journal.pone.0206582 (PMC6211711; doi:10.1371/journal.pone.0206582)
Supplement: S3 Model description — (DOC) [file pone.0206582.s003.doc]

**Model description: Segmented nonlinear mixed-effects model for the number of inflorescences per plant**

The non-linear mixed effects model implemented in this study (Baty et al. 2015) is:

$F_{ik}(t_{k})=\phi_{ik}(1-e^{-(t_{k}-\Lambda_{k})/\mu_{ik}})\mathbf{1}_{(\Lambda_{k},\infty)}(t_{k})$, (1)

where $F_{ik}(t{}_{k})$ is the number of inflorescences at time $t_{k}$ for plant *i* in the sowing time group *k*; $\Lambda_{k}$ is the lag or resting time with no inflorescences for group *k* (defined as the last week in which inflorescences of plants from that group were still absent), and $\mathbf{1}_{(\Lambda_{k},\infty)}(t_{k})$ is the indicator function, taking the value 1 if *t_k_* > $\Lambda_{k}$, and 0 otherwise. The value of $\Lambda_{k}$ was assumed to be known *a priori*. However, as the week indicating the resting time did not coincide for all plants in the same sowing group, its given value was the one shared by more than 75% of the plants observed in that particular group. Under this criterion, plants not fulfilling the condition were removed before model fitting. Thus, $\phi_{ik}$ and $\mu_{ik}$ were the only estimated parameters in the models: $\phi_{ik}$ is the peak parameter indicating the maximum number of inflorescences (a plateau reached asymptotically), and $\mu_{ik}>0$ is the rate of change governing the steepness of the curve for the number of inflorescences as time *t* elapses: larger values of $\mu_{ik}$ correspond to steeper curves. Each $\varepsilon_{ik}$ is the random error term of the non-linear model, $\varepsilon_{ik}\sim N(\mathbf{0},\sigma_{k}^{2})$. Therefore, we assume that the segmented model is an approximation for the description of a discrete variable (number of inflorescences), using an expression for a continuous variable. The fixed and random effects for $\phi_{ik}$ and $\mu_{ik}$in (1) determine two vectors $\mathbf{\beta}_{k}$ and$\mathbf{b}_{ik}$:

$\left[\begin{matrix} \phi_{ik} \\ \mu_{ik} \end{matrix}\right]=\mathbf{\beta}_{k}+\mathbf{b}_{ik}=\left[\begin{matrix} \beta_{\phi_{k}} \\ \beta_{\mu_{k}} \end{matrix}\right]+\left[\begin{matrix} b_{\phi_{ik}} \\ b_{\mu_{ik}} \end{matrix}\right]$, $\mathbf{b}_{ik}\sim N(\mathbf{0},\Psi_{k})$ *k*=1,2,3. (2)

Here $\mathbf{\beta}_{k}$, *k*=1, 2, 3, is the fixed effects vector constituted by the mean peak parameter ($\beta_{\phi_{k}}$) and the mean rate of change ($\beta_{\mu_{k}}$). The random effects vectors (**b***_ik_*) represent the deviation of the unknown parameters in (1) from their corresponding population mean; they are assumed to be independent for different plants and sowing times, and the random errors (*ε_ijk_*) in (1) are independent for different sowing times, as well as being independent of the random effects. Calculations of the estimated effects were carried out in the R-package nlme (Pinheiro and Bates 2000). In order to guarantee convergence, plants whose inflorescence count varied erratically were removed from analysis. Model (1) with the decomposition (2) was assumed as the maximal model. Given that each fitted model depends on a pre-established resting time $\Lambda_{k}$, only 95% confidence intervals for $\beta_{\phi_{k}}$and $\beta_{\mu_{k}}$ were calculated for comparison. The predicted values of $\hat{\mu}_{ik}$ were used to estimate the *mean flowering time* ($MFT_{ik}$) of plant *i* sown at time *k* (Bell *et al.*, 2001), defined as:

$MFT_{ik}=\Lambda_{k}+\hat{\mu}_{ik}$. (3)

The *MFT* is interpreted as the time needed for a plant to attain a number of inflorescences near the 63 % of the asymptotic peak $\hat{\phi}_{ik}$. This can be proved by simple substitution of $t_{ik}=\Lambda_{k}+\hat{\mu}_{ik}$ in the expression for the predicted number of inflorescences$\hat{y}_{ik}$ given in model (1). To compare the sowing times of plants with respect to their average *MFT*, an ANOVA was performed, followed by a Tukey’s test; R was used for the calculations (R Core Team, 2017).

**References**

Bell, C., Paterson, D.H., Kowalchuk, J.M., Padilla, J., Cunningham, D.A., 2001. A comparison of modelling techniques used to characterise oxygen uptake kinetics during the on-transient of exercise. Exp. Physiol. 86, 667–676.

Baty, F., Ritz, C., Charles, S., Brutsche, M., Flandrois, J., Delignette-Muller, M., 2015. A Toolbox for Nonlinear Regression in R: The Package nlstools. J. Stat. Softw. 66, 1-21.

Pinheiro, J.C., Bates, D., 2000. Mixed-Effects Models in S and S-PLUS. Springer: New York

R Core Team. 2017. R: A language and environment for statistical computing. R Foundation for Statistical Computing, Vienna, Austria.
