# Supplementary material for: The effect of sowing time on the growth of chia (Salvia hispanica L.): What do nonlinear mixed models tell us about it?
Source: PLoS One. 2018 Nov 1;13(11):e0206582. doi: 10.1371/journal.pone.0206582 (PMC6211711; doi:10.1371/journal.pone.0206582)
Supplement: S1 Fig — (DOC) [file pone.0206582.s004.doc]

**Plots of fitted and predicted responses of chia plants using nonlinear mixed-effects models**

**A. Plot of fitted and predicted Richards curves for height of chia plants**

**
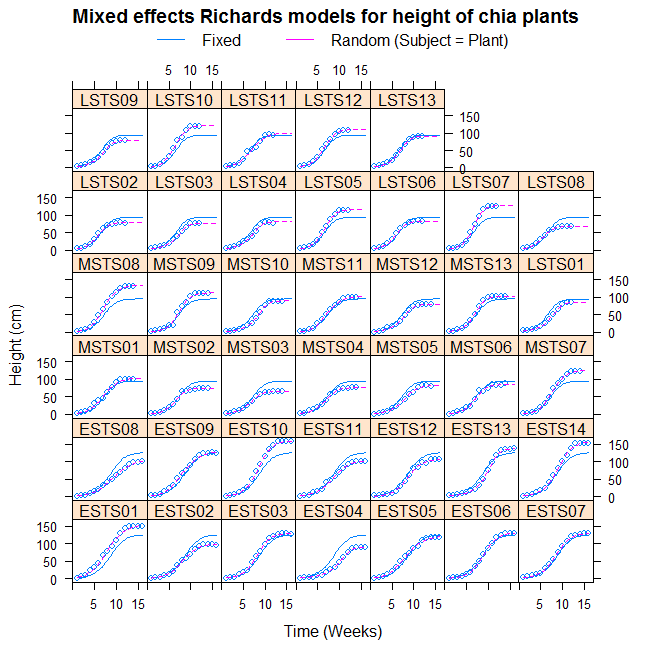
**

Figure 1. Fitted and predicted four-parameter Richards models for the height of chia plants classified by sowing time: EST = early, MST = medium, LST = late. Each plant is identified by the letter S (subject) and the number of plant within the sowing time group.

**B. Plot of fitted and predicted Double Richards curves for leaves of chia plants**


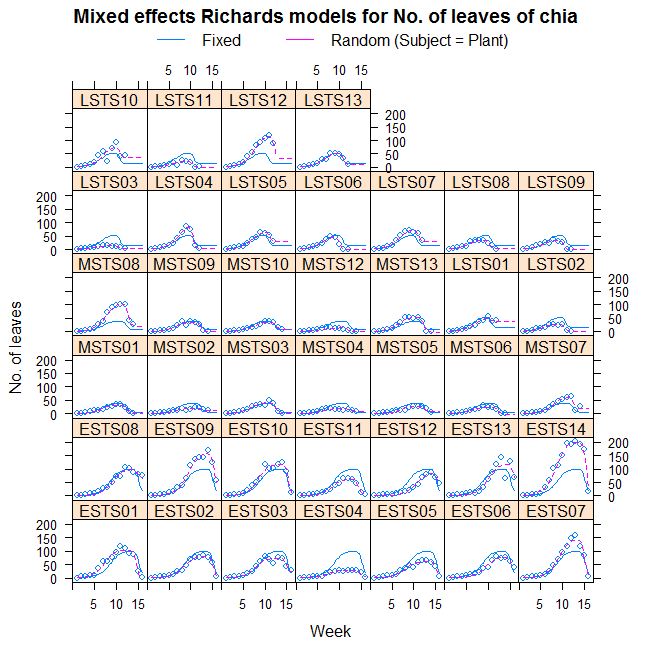


Figure 2. Fitted and predicted Double Richards models for the height of chia plants classified by sowing time: EST = early, MST = medium, LST = late. Each plant is identified by the letter S (subject) and the number of plant within the sowing time group.

**C. Plots of fitted and predicted non-linear mixed-effects segmented models for number of inflorescences of chia plants**


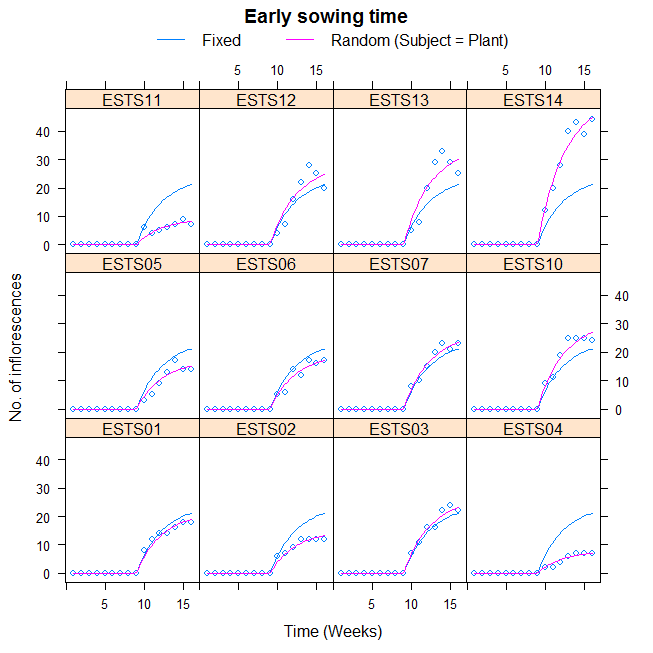


Figure 3. Fitted and predicted non-linear mixed-effects segmented models for plants belonging to the early sowing time (EST) group, sharing the same value of the parameter indicating the lag or resting time with no inflorescences, $\Lambda_{1}=9$. Plants are also identified by the letter S (subject) followed by the number of plant. Plants 08 and 09 were outliers, removed from the analysis.


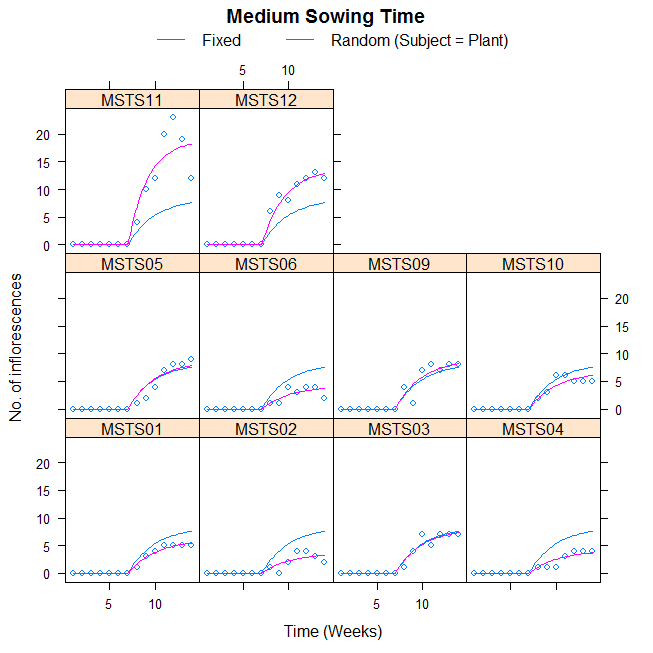


Figure 4. Fitted and predicted non-linear mixed-effects segmented models for plants belonging to the medium sowing time (MST) group, sharing the same value of the parameter indicating the lag or resting time with no inflorescences, $\Lambda_{2}=7$. Plants are also identified by the letter S (subject) followed by the number of plant. Plants 07, 08 and 12 were outliers, removed from the analysis.


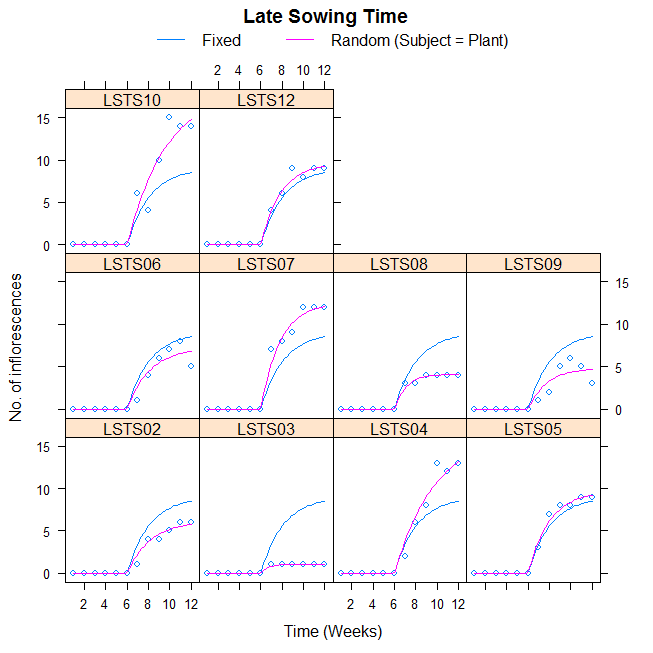


Figure 5. Fitted and predicted non-linear mixed-effects segmented models for plants belonging to the late sowing time (LST) group, sharing the same value of the parameter indicating the lag or resting time with no inflorescences, $\Lambda_{3}=6$. Plants are also identified by the letter S (subject) followed by the number of plant. Plants 01 and 11 were outliers, removed from the analysis.
